# The Veterans Aging Cohort Study (VACS)‐National: A Real‐World Data Source Profile

**DOI:** 10.1002/pds.70465

**Published:** 2026-08-23

**Authors:** Christopher T. Rentsch, Vincent Lo Re, Melissa Skanderson, Kathleen A. McGinnis, Farah Kidwai‐Khan, Charles Alcorn, Anne C. Black, Kristina Crothers, Vincent C. Marconi, Matthew J. Freiberg, Jessie Torgersen, Amy C. Justice, William C. Becker, Lesley S. Park

**Affiliations:** ^1^ Faculty of Epidemiology and Population Health London School of Hygiene & Tropical Medicine London UK; ^2^ Department of Internal Medicine Yale School of Medicine New Haven Connecticut USA; ^3^ VA Connecticut Healthcare System, Department of Veterans Affairs West Haven Connecticut USA; ^4^ Center for Pharmacoepidemiology and Treatment Science, Rutgers Institute for Health Health Care Policy and Aging Research New Brunswick New Jersey USA; ^5^ Department of Biostatistics School of Public Health, University of Pittsburgh Pittsburgh Pennsylvania USA; ^6^ Department of Medicine VA Puget Sound Health Care System and University of Washington Seattle Washington USA; ^7^ Department of Veterans Affairs Atlanta Veterans Affairs Medical Center Decatur Georgia USA; ^8^ Division of Infectious Diseases Emory University School of Medicine Atlanta Georgia USA; ^9^ Department of Global Health Rollins School of Public Health, Emory University Atlanta Georgia USA; ^10^ Department of Medicine Vanderbilt Health and Veteran Affairs TVHS Nashville Nashville Tennessee USA; ^11^ Department of Medicine Perelman School of Medicine, University of Pennsylvania Philadelphia Pennsylvania USA; ^12^ Department of Biostatistics, Epidemiology, and Informatics, Center for Clinical Epidemiology and Biostatistics, Center for Real‐World Effectiveness and Safety of Therapeutics Perelman School of Medicine, University of Pennsylvania Philadelphia Pennsylvania USA; ^13^ Department of Health Policy and Management Yale School of Public Health New Haven Connecticut USA; ^14^ Division of Epidemiology University of California Berkeley School of Public Health Berkeley California USA

**Keywords:** aging, comparative effectiveness research, drug safety, electronic health records, medication utilization, pharmacoepidemiology, veterans

## Abstract

The Veterans Aging Cohort Study (VACS)‐National is a large, longitudinal real‐world data resource derived from electronic health records within the Department of Veterans Affairs (VA), the largest integrated healthcare system in the United States. As of December 2025, VACS‐National includes > 14.9 million Veterans who have received care in the VA since 2000. It captures information on VA‐related outpatient and inpatient care and includes structured (e.g., diagnostic codes, laboratory results, vital signs, dispensed medical products), semi‐structured (e.g., radiology and pathology reports) and unstructured (e.g., progress notes) fields, collected from over 1300 points of care nationwide. Its longitudinal structure supports extended follow‐up across demographically and clinically diverse populations within an integrated healthcare system. Key features for pharmacoepidemiological research include near real‐time availability of data on dispensed medications, including biologics, vaccines, and devices; routinely‐collected laboratory measurements; and computable phenotypes, including validated natural language phenotypes. The resource includes linkages to Centers for Medicare and Medicaid Services (CMS) data, VA Community Care data, National Death Index, and deeply phenotyped VACS sub‐cohorts with an extensive library of biomarkers and survey data. VACS‐National has supported a wide range of research, including drug utilization, drug safety, comparative effectiveness, and epidemiologic and health outcomes research. Strengths include its national scale, longitudinal follow‐up, and rich clinical record; limitations include a small proportion of women and potential incomplete capture of care outside the system or not recoverable through linked datasets. VACS‐National provides a comprehensive platform for generating real‐world evidence to inform clinical, regulatory, and public health decision‐making.

## Five Key Points

- VACS‐National is a large, longitudinal electronic health record resource including > 14.9 million Veterans receiving care in the largest integrated healthcare system in the US.
- It captures all outpatient and inpatient care received and includes many validated phenotypes.
- The resource enables longitudinal follow‐up across demographically and clinically diverse populations, including evaluation of utilization, safety, and effectiveness of medical products.
- Detailed data on dispensed medical products in outpatient and inpatient settings support precise exposure definitions for pharmacoepidemiologic research.
- Linkages to external data sources and deeply phenotyped sub‐cohorts extend its utility for validation and translational research.

## 1 Introduction

The Veterans Aging Cohort Study (VACS)‐National is a large, longitudinal real‐world data resource derived from electronic health records (EHR) within the Department of Veterans Affairs (VA), the largest integrated healthcare system in the United States (US). Built on over two decades of infrastructure developed through the VACS research network, VACS‐National encompasses the full population of Veterans who have accessed VA care, supporting a wide range of pharmacoepidemiologic and clinical research applications.

This paper describes the structure, contents, and research applications of VACS‐National to inform its appropriate use for generating real‐world evidence.

## 2 Overview of the Data Source

VACS‐National includes all Veterans honorably discharged from the US military who access care within the VA healthcare system, with longitudinal data available from 2000 onward. The database captures care delivered across the full spectrum of VA services, including outpatient, inpatient, and ancillary care, and supports the construction of dynamic cohorts with extended follow‐up. VACS‐National is updated annually to incorporate new patients, while underlying data domains (described below) are refreshed nightly, supporting timely ascertainment of exposures and outcomes.

The VA has over 1300 points of care nationwide, including hospitals, medical centers, and community‐based outpatient clinics, capturing detailed clinical information through a unified EHR infrastructure. This integrated system enables comprehensive longitudinal follow‐up across both outpatient and inpatient settings and near real‐time evaluation of medical product utilization, safety, and effectiveness. VA data have also been used in regulatory contexts, including post‐authorization safety studies conducted in collaboration with regulatory agencies [1, 2].

VACS originated in 1998 with VACS‐HIV, its marquee cohort of people with HIV in the VA, alongside demographically, geographically, and temporally matched comparators without HIV [3, 4]. Over two decades, the program developed and validated a wide range of computable phenotypes (encompassing structured, semi‐structured, and unstructured data), data curation and transformation pipelines, and analytic approaches using VA EHR data. VACS‐National was established in 2020 to extend these methods to the full population of Veterans receiving care in the VA. The VACS research network thus comprises related cohorts and analytic datasets that share common underlying data sources, code, and variable definitions, enabling consistent and reproducible research across studies.

The VA Corporate Data Warehouse (CDW) is a national repository that integrates clinical and administrative data across the VA with patient‐level data retaining clear provenance to their source systems [5, 6]. Patient‐level data are extracted, harmonized, and transformed into research‐ready datasets using standardized definitions and reusable code. Data access and analyses are conducted within the VA Informatics and Computing Infrastructure (VINCI) [7], a secure computing environment for approved research, with access subject to regulatory approvals, data use agreements, and secure data handling requirements. Data are also available in the Observational Medical Outcomes Partnership (OMOP) Common Data Model, supporting standardized analyses and cross‐institutional collaboration.

## 3 Population Characteristics and Representativeness

The intentionally broad inclusion criterion for VACS‐National, which currently encompasses > 14.9 million Veterans, allows for the construction of study‐specific populations, as individual studies typically apply additional inclusion and exclusion criteria tailored to specific research objectives.

**FIGURE 1:**
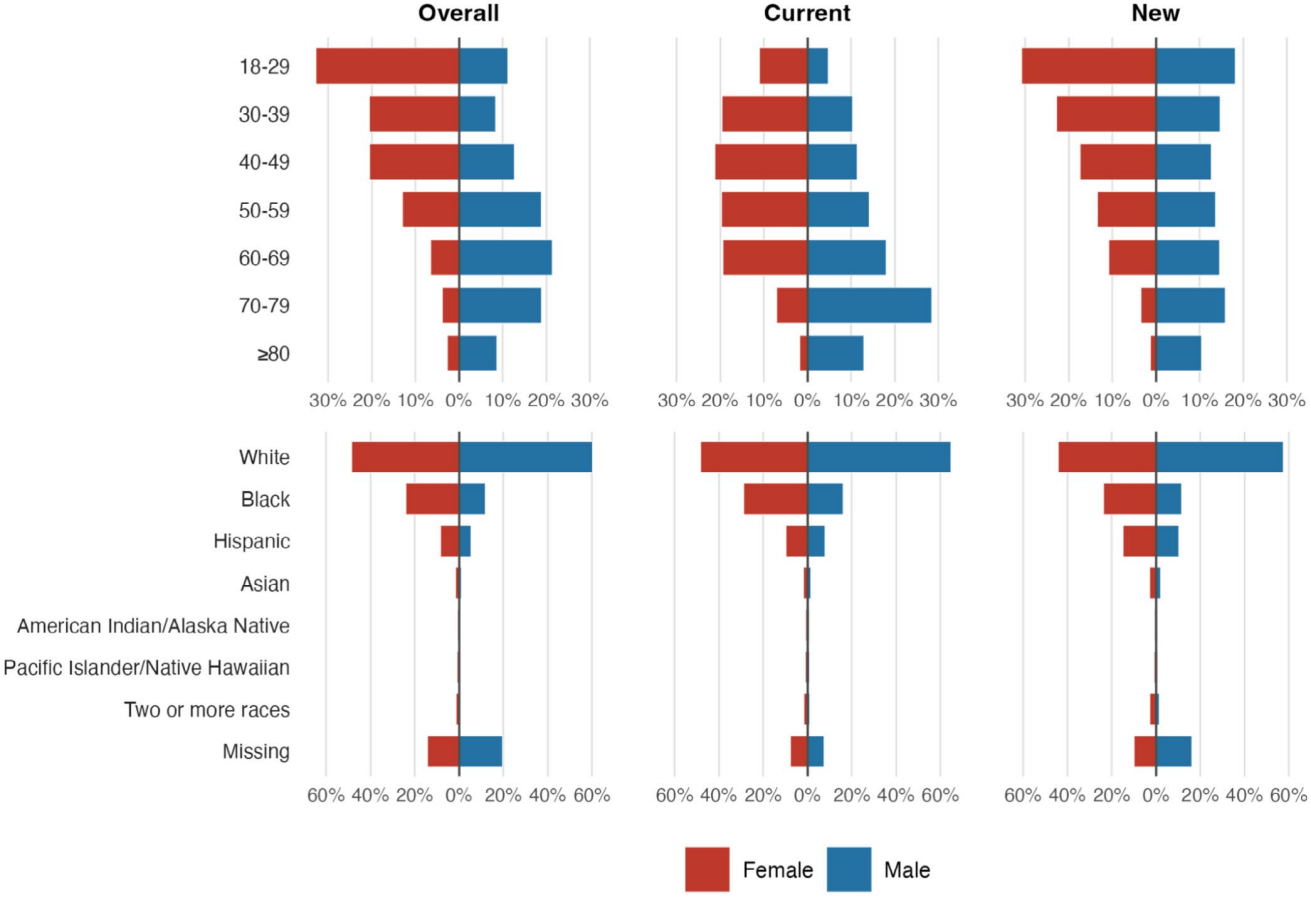
Distribution of age, race, and ethnicity by sex in VACS‐National. *Note:* Bars show percentage of females (red) and males (blue). Overall patients are those with any activity between 1 Jan 2000 and 31 Dec 2025. Current patients are those with any activity between 1 Jan 2024 and 31 Dec 2025. New patients are those whose first visit is between 1 Jan 2024 and 31 Dec 2025. Age is as of first visit for overall and new patients, and as of 1 Jan 2024 for current patients.

The population is predominantly male (92.3%), with age distribution skewed toward older adults. Women represent a growing proportion of Veterans in care, encompassing 7.7% (*n* = 1 141 074) of the overall cohort and 11.3% (*n* = 778 218) of currently active patients, as of 31 December 2025 (Table 1, Figure 1). Racial and ethnic diversity is substantial, with a wide range of socioeconomic contexts and geographic settings, enabling investigation of disparities in health outcomes and pharmacoequity [8] in treatment patterns.

**TABLE 1:** Characteristics of patients included in the Veterans aging cohort study (VACS)‐National.

	Overall	Current	New
Number of patients, *n*	14 922 108	6 878 550	624 761
Sex			
Female	1 141 074 (7.7)	778 218 (11.3)	79 650 (12.8)
Male	13 781 034 (92.3)	6 100 332 (88.7)	545 111 (87.2)
Age, years			
18–29	1 915 368 (12.8)	376 212 (5.5)	123 682 (19.8)
30–39	1 390 000 (9.3)	784 723 (11.4)	98 008 (15.7)
40–49	1 987 156 (13.3)	859 997 (12.5)	82 951 (13.3)
50–59	2 743 913 (18.4)	1 014 622 (14.8)	84 945 (13.6)
60–69	3 016 736 (20.2)	1 251 761 (18.2)	87 867 (14.1)
70–79	2 642 609 (17.7)	1 791 407 (26.0)	89 221 (14.3)
≥ 80	1 226 326 (8.2)	799 828 (11.6)	58 087 (9.3)
Race and ethnicity			
White	8 859 913 (59.4)	4 325 325 (62.9)	367 778 (58.9)
Black	1 899 303 (12.7)	1 204 146 (17.5)	85 839 (13.7)
Hispanic	847 497 (5.7)	560 206 (8.1)	71 581 (11.5)
Asian	153 675 (1.0)	101 592 (1.5)	13 787 (2.2)
American Indian/Alaska Native	81 226 (0.5)	46 019 (0.7)	3909 (0.6)
Pacific Islander/Native Hawaiian	96 204 (0.6)	54 314 (0.8)	4275 (0.7)
Two or more races	104 726 (0.7)	67 150 (1.0)	10 292 (1.7)
Missing	2 879 564 (19.3)	519 798 (7.6)	67 300 (10.8)
Census region			
Midwest	3 255 313 (21.8)	1 423 350 (20.7)	110 180 (17.6)
Northeast	2 132 255 (14.3)	856 521 (12.5)	70 153 (11.2)
South	6 382 331 (42.8)	3 095 841 (45.0)	302 340 (48.4)
West	3 152 209 (21.1)	1 502 838 (21.8)	142 088 (22.7)
Residence type			
Rural	5 078 498 (34.0)	2 356 903 (34.3)	196 251 (31.4)
Urban	9 843 610 (66.0)	4 521 647 (65.7)	428 510 (68.6)
Year of first VA visit			
2000–2005	7 166 825 (48.0)	2 109 211 (30.7)	—
2006–2010	2 303 014 (15.4)	1 165 950 (17.0)	—
2011–2015	2 244 366 (15.0)	1 276 921 (18.6)	—
2016–2020	1 716 341 (11.5)	1 056 055 (15.3)	—
2021–2025	1 491 562 (10.0)	1 270 413 (18.5)	624 761 (100.0)

*Note:* Reported as *n* (%). Overall patients are those with any activity between 1 Jan 2000 and 31 Dec 2025. Current patients are those with any activity between 1 Jan 2024 and 31 Dec 2025. New patients are those whose first visit is between 1 Jan 2024 and 31 Dec 2025. Age is as of first visit for overall and new patients, and as of 1 Jan 2024 for current patients.

Abbreviation: VA, Department of Veterans Affairs.

Veterans in VACS‐National have a high burden of chronic medical conditions, including hypertension, diabetes, chronic kidney disease, liver disease, chronic lung diseases, cancer, coronary heart disease, heart failure, ischemic stroke, and mental health conditions including depression, post‐traumatic stress disorder, and substance use disorders [9, 10]. This combination of medical, mental health, and behavioral comorbidity makes VACS‐National particularly well suited to pharmacoepidemiology studies of aging and complex patient populations.

VACS‐National supports extended follow‐up through its open cohort design, with an average follow‐up of approximately 10.2 years (interquartile range 3.8–17.2 years), allowing ongoing inclusion of new patients while maintaining longitudinal observation of existing ones.

Absolute measures of disease incidence, prevalence, and risk may differ from the general population due to underlying differences in demographics and comorbidity burden. However, a prior report has shown that, after accounting for characteristics typically adjusted for in analyses—including age, sex, race, ethnicity, geographic region, and rural or urban residence—there is no difference in total disease burden between Veterans and non‐Veterans [11]. Systematic reviews and other reports comparing VA and non‐VA care have also shown that clinical quality and safety within the VA are generally similar to or better than those in non‐VA settings [12, 13]. VACS‐National is therefore well suited for estimating both absolute and relative treatment effects and associations, though absolute measures should be interpreted in the context of the Veteran population's demographic and clinical characteristics. This has been demonstrated across multiple research contexts, including studies of COVID‐19 outcomes [14] and longstanding work in cohorts of people with HIV and/or hepatitis C where results have been consistent with large international collaborations [15, 16, 17, 18, 19].

## 4 Data Domains

### 4.1 Clinical Encounters, Diagnoses, and Procedures

Clinical data include detailed information on outpatient visits, hospitalizations, and procedures, captured routinely as part of care delivery across the VA. Community care encounters authorized and reimbursed by the VA through the Community Care program (previously referred to as fee‐basis) and expanded under the VA MISSION Act of 2018 are additionally captured, enabling more complete ascertainment of care received outside VA facilities [20]. Diagnoses are recorded using International Classification of Diseases (ICD‐9/10) codes, and procedures using standard coding systems, including Current Procedural Terminology (CPT) and Healthcare Common Procedure Coding System (HCPCS), enabling the construction of fixed‐time point and longitudinal disease histories.

### 4.2 Pharmacy and Medical Products Data

Data on prescribed and dispensed medications in outpatient and inpatient settings are derived from VA pharmacy records, governed by VA Pharmacy Benefits Management Services' national formulary [21]. Outpatient medications are standardized using RxNorm [22], enabling consistent classification across therapeutic classes, while inpatient medication administration is captured through the Bar Code Medication Administration (BCMA) system [23, 24], supplemented by intravenous and unit dose dispensing records from pharmacy data. Unlike many data sources that capture prescriptions alone, VA data include detailed dispensing information, including prescribed and dispensed dates, quantity dispensed, days' supply, dose, and directions for use (“sig”). Vaccine data are captured through CDW immunization tables and pharmacy dispensing records, with CVX codes available for all vaccines dispensed by VA pharmacies, including those prescribed by outside providers. Capture may be incomplete for vaccines that do not require a pharmacy order or for vaccines received outside the VA. Medications and vaccines dispensed through VA Community Care are also captured, further extending coverage of care received outside VA facilities. For COVID‐19 vaccines, capture was extended to include vaccines administered outside the VA through linkage to national pharmacy databases and provider‐recorded self‐report [25]. Biologic therapies administered by injection or infusion are similarly captured through VA pharmacy dispensing and infusion administration records [26]. Implanted medical devices, such as joint prostheses [27, 28], are identifiable through diagnostic and procedure codes within the CDW. These data support the construction of precise exposure definitions and longitudinal treatment histories, which are critical for pharmacoepidemiologic analyses.

### 4.3 Laboratory Data and Vital Signs

Laboratory tests and clinical measurements are routinely captured and reported. In contrast to healthcare systems where laboratory testing may be performed in response to clinical concerns, Veterans undergo routine laboratory testing as part of annual health assessments, resulting in high availability of longitudinal laboratory data. Validation studies have demonstrated strong concordance between VACS and OMOP‐based laboratory data extraction approaches, supporting the reliability of VA laboratory data [29]. Commonly available measures include markers of renal function, liver injury, hematologic status, and metabolic health. Vital signs including height, weight, and blood pressure are recorded longitudinally.

### 4.4 Mortality

Mortality data are available from the Death Ascertainment File, which is updated monthly and draws on multiple sources. Ascertainment is highly complete and timely because VA benefits administration is tied to death reporting; accuracy has been shown to be consistent with the National Death Index [30, 31]. Information on cause of death is available through linkage with the National Death Index, although these data are subject to a longer reporting lag compared with all‐cause mortality.

### 4.5 Health Behaviors and Patient‐Reported Measures

VACS‐National includes repeated measures of routinely measured key health behaviors and patient‐reported outcomes. These include alcohol use, assessed using validated instruments such as the Alcohol Use Disorders Identification Test‐Concise (AUDIT‐C) [32, 33, 34], and smoking status [35, 36], both of which are recorded longitudinally and support the study of behavioral trajectories over time. In addition, mental health measures, including standardized instruments such as the Patient Health Questionnaire [37, 38] and Generalized Anxiety Disorder [39] scale, are available for many patients, enabling investigation of mental health comorbidity. Pain intensity is routinely assessed using the numeric rating scale [40, 41], a standardized patient‐report measure that is recorded longitudinally in clinical practice.

### 4.6 Semi‐Structured, Unstructured, and Imaging Data

In addition to structured data domains, VACS‐National includes semi‐structured (e.g., radiology, pathology reports), unstructured (e.g., clinical notes, discharge summaries), and imaging (e.g., radiology, pathology) data. Data are available through the CDW and have been used in natural language processing (NLP) analyses, including large language models, to identify symptoms, adverse events, and clinical phenotypes not easily captured through structured data alone [42, 43, 44, 45]. Medical imaging reports have been used in NLP analyses to identify clinically significant findings. Digital images, such as CT scans and pathology slides, are available for radiomics and deep learning analyses, although processing these data at scale requires additional resources compared with other data domains. Reusable pipelines for creating AI‐ready data, incorporating strategies to reduce algorithmic bias and promote fairness, have been developed to support future applications [46]. The VA offers substantial computing resources in partnership with the Department of Energy to support large‐scale imaging processing and analysis.

### 4.7 Linkages to Other Data Sources

VACS‐National is linked to several external and specialized data sources that extend its utility for research. Linkage to Centers for Medicare and Medicaid Services (CMS) data is commonly used to capture care and outpatient medications prescribed through Part D received outside the VA, although these data are subject to a reporting lag. Other linkages are available, including cancer registries (e.g., VA Cancer Registry) [47, 48], surgical outcomes data (e.g., VASQIP) [49, 50, 51], and geospatial and neighborhood‐level data, including the Area Deprivation Index (ADI) [52], linked via residential address to support investigation of social determinants of health and environmental exposures; use depends on specific study approvals.

VACS‐National is linked to deeply phenotyped sub‐cohorts within the VACS program. These include repeated survey cohorts with patient‐reported behaviors and outcomes [4], biomarker cohorts with detailed multi‐omic and inflammatory biomarkers that have been used to develop pharmacogenomics‐informed clinical decision support tools [53], and specialized studies that include biomarkers of substance use [36, 54, 55, 56]. These linkages are frequently used to develop, refine, and validate EHR‐based phenotyping algorithms.

## 5 Data Structure and Key Variables

VACS‐National is organized around a unique patient identifier that links across domain‐specific datasets, including pharmacy, encounters, diagnoses, procedures, laboratory results, vital signs, and mortality. These can be assembled into analytic datasets with time‐updated information on exposures, outcomes, and covariates, supporting a wide range of longitudinal study designs.

### 5.1 Computable Phenotypes

VACS develops and maintains a range of computable phenotypes using multi‐domain VA EHR data (Table 2). Such phenotypes capture complex chronic disease manifestations [57, 58, 59], longitudinal substance use trajectories [60, 61, 62], and surgical interventions [27]. Automated text extraction and NLP algorithms have been developed for analysis of unstructured text to accurately identify pulmonary function tests, hepatic steatosis, and osteoporosis [42, 44, 45]. Deep learning tools applied for medical digital image analysis demonstrated reliable and accurate identification of clinically significant phenotypes [43]. The application of these approaches to a national cohort facilitates comprehensive evaluation of medication exposures and outcomes associated with such phenotypes.

**TABLE 2:** Select computable phenotypes available to researchers using VACS‐National.

Phenotype	Reference PMID (s)
Abdominal radiology (liver masses, ascites, varices)	21622934
Alcohol (screening, history, daily consumption, hazardous/harmful use, use disorder, trajectories)	23050632, 32971391, 18432391, 16849969, 28295416, 32034291
Antiretroviral therapy (timing, regimen)	23836591, 22337823
Bone mineral density, osteoporosis	39526751
Cancer	25580366
Charlson Comorbidity Index	38356736
Chronic obstructive pulmonary disease	29923258
Cigarette smoking (current status, trajectories)	17503106, 21911825, 37252267, 28492393
Coronary artery disease	16849969
Diabetes	19444074, 33006179
Direct‐acting antiviral therapy (timing, regimen)	30195616
Falls, serious falls, fractures, fragility fractures	26567329, 36094483, 32860222, 21063542, 21359191
Heart failure, congestive	16849969
Hepatic decompensation	21626605, 24723077, 39477692
Hepatic steatosis	37276449, 38896066
Hepatitis C virus	16849969
Hepatotoxicity Medication Score	39662972
Hip periprosthetic joint infection	34170057, 41490605
Hispanic ethnicity	36893404
Human immunodeficiency virus status	16849965, 16849964
Hypertension	24065316
Incarceration	31095055
Macular degeneration	21139706
Myocardial infarction	24065316, 23459863
Oral hypoglycemic fill/refill adherence	21779543, 20388863
Palliative care	29254358
Pancreatitis	16849969
Peripheral vascular disease	16849969
Pneumonia, community‐acquired	32724760
Prescription opioids (receipt, trajectories)	31317364
Pulmonary function tests	31945115
Quality of care/performance	17279023
Socially sensitive behaviors	18694046
Stroke, transient ischemic attack	16849969
VACO Index	33175863, 34583962
VACS Index	24226058, 24667813, 31565954, 19106740
VACS‐CCI Index	38356736

Abbreviations: CCI, Charlson Comorbidity Index; PMID, PubMed Identifier; VACO, Veterans Health Administration COVID‐19; VACS, Veterans Aging Cohort Study.

The use of shared algorithms across studies enhances reproducibility and supports consistent measurement of exposures and outcomes within VACS‐National. These algorithms are publicly accessible through the Centralized Interactive Phenomics Resource (CIPHER), a VA‐wide knowledgebase that standardizes the documentation, sharing, and reuse of EHR‐based phenotype definitions [63].

### 5.2 Derived Variables and Risk Scores

VACS‐National has supported the construction and implementation of derived variables and validated risk scores. Notably, the VACS Index 2.0 is a widely used measure of physiologic frailty that incorporates age and routinely collected laboratory markers to predict mortality risk [64]. The VACS‐Charlson Comorbidity Index (VACS‐CCI) adds a widely used measure of comorbidity burden to the VACS Index, which has been shown to moderately improve discrimination and calibration of 10‐year mortality [65]. The Veterans Health Administration COVID‐19 (VACO) Index was developed to estimate risk of mortality after SARS‐CoV‐2 infection [66, 67]. The Hepatotoxicity Score was developed to adjust for concomitant hepatotoxic medication exposure within hepatic safety studies [68]. These indices have been externally validated in non‐VA populations and are used in clinical and research settings through MDCalc and have been incorporated into at least eight other healthcare systems using the Epic platform to date [69, 70, 71].

## 6 Data Refresh, Updates, and Timeliness

The VA CDW is updated nightly, enabling near real‐time assessment of exposures and outcomes. For most studies, investigators use curated VACS‐National datasets that are updated annually, with the cycle reflecting the time needed to ensure data completeness and quality across all domains rather than a limitation of underlying data availability. The annual cohort update incorporates new patients, extends follow‐up for existing patients, and re‐runs standardized data cleaning pipelines and phenotyping algorithms across the full cohort to ensure consistency and completeness of derived variables. These versioned datasets, reflecting the time needed to ensure data completeness and quality, provide a stable and reproducible analytic environment. For studies requiring more immediate data, such as those addressing emerging exposures or outcomes, investigators may work directly with the daily‐updated underlying CDW data, though additional processing and validation may be required.

Despite the near real‐time availability of many core data elements, reporting completeness varies across data sources. Some administratively derived outcomes may require 30 days or longer for full completeness. Mortality data from the Death Ascertainment File are updated monthly, whereas cause of death from the National Death Index is subject to a lag of approximately 2 years. Linkages to CMS data are similarly subject to a lag of 1–2 years.

## 7 Comparison With Other Data Sources

VACS‐National complements other commonly used real‐world data sources. Compared with administrative claims data, which are primarily designed for billing purposes, VACS‐National includes detailed clinical information (e.g., laboratory results, vital signs, patient‐reported measures) and comprehensive pharmacy dispensing data. In contrast to many employer‐based or regional integrated healthcare systems, VACS‐National provides nationwide coverage and supports extended longitudinal follow‐up within a single integrated healthcare system as well as “out‐of‐network” coverage through CMS and VA Community Care. Compared with disease‐specific registries, VACS‐National provides broader population coverage and supports the study of multiple conditions and treatments within the same resource, while still enabling linkage to deeply phenotyped sub‐cohorts within the VACS program. More broadly, a distinction should be made between a data source and a curated cohort derived from that source. While individual studies may extract and process VA data independently for each project, the VACS team's approach of maintaining a consistently defined, regularly updated cohort with standardized variable definitions and reusable code promotes greater reproducibility and comparability across studies.

## 8 Strengths

VACS‐National has several key strengths including its scale as one of the largest integrated EHR systems available for research with longitudinal follow‐up across both outpatient and inpatient settings within a unified system. The detailed dispensing data on pharmacy and other medical products support precise exposure definitions allowing for accurate identification of inpatient and outpatient medications. Routinely collected laboratory and clinical measurements enable laboratory‐based outcomes and risk scores. Repeated measures of health behaviors and patient‐reported outcomes documented in the EHR support trajectory analyses. Computable phenotypes leveraging multiple EHR domains, including NLP analysis of unstructured text and use of deep learning tools for direct medical image analysis, allow for standardized variable definitions across studies that enhance reproducibility. Linkage to external data sources and deeply phenotyped sub‐cohorts further extend the scope of the resource.

## 9 Limitations

VACS‐National has limitations for consideration when interpreting findings. The population demographics differ from the general US population, which may affect the generalizability of absolute risk estimates. Ascertainment of some exposures, outcomes, and covariates for some patients may be incomplete; linkage to CMS and Community Care addresses this gap. As with all EHR data, coding practices or incomplete documentation may result in misclassification. As in other real‐world settings, data completeness may vary across data domains and clinical settings, and some elements such as over‐the‐counter medication and patient‐reported measures may be variably recorded depending on care context. Changes in coding systems (ICD‐9–10) and clinical practice may affect the consistency of certain variables across calendar periods. Finally, linked data sources (e.g., National Death Index and CMS claims) are subject to reporting lags.

## 10 Considerations for Researchers

Several practical considerations are important when designing studies using VACS‐National. Medication exposure definitions should be based on dispensing records, and investigators should recognize that dispensing does not fully capture adherence although it does allow for calculation of adherence proxies including proportion of days covered and medication possession ratio [72, 73]. As in all real‐world data sources, sensitivity analyses to quantify the potential impact of residual bias are a hallmark of good pharmacoepidemiologic practice and should be considered a routine component of designing a study [74], with external validation additionally warranted depending on the research question. Confounding by indication and high comorbidity burden are common in this population, and appropriate design and analytic approaches, such as active comparator new‐user designs and propensity score methods, should be considered. Where ascertainment of non‐VA care is important, linkage to CMS and Community Care data should be used, with attention to the associated reporting lag when defining study periods. Temporal changes in coding practices and data availability should be accounted for in exposure and outcome definitions, particularly for studies spanning the ICD‐9 to ICD‐10 transition. Finally, investigators should consider the structure and completeness of specific data elements relevant to their study and consult available phenotyping algorithms where possible.

## 11 Data Access

Due to VA regulations and data governance requirements, analytic datasets derived from VACS‐National are not permitted to leave the VA secure computing environment without an approved data use agreement. However, VA data are made available to researchers with an approved VA study protocol. Access requires appropriate regulatory approvals, including VA research and development review, and must be conducted within secure environments such as VINCI. For more information, please visit https://www.virec.research.va.gov. Researchers interested in discussing potential collaborations or research opportunities using VACS‐National may contact the VACS team at vacs@yale.edu or visit https://medicine.yale.edu/internal‐medicine/vacs/.

## 12 Example Pharmacoepidemiologic Research Outputs

VACS‐National has supported a wide range of pharmacoepidemiologic applications across therapeutic areas and methodological approaches. These include studies of drug safety and comparative effectiveness [75], including analyses of COVID‐19 vaccinations, treatments, and outcomes [14, 25, 76, 77, 78, 79]. The resource has supported drug repurposing efforts by integrating EHR data with genetic and clinical prioritization approaches, including investigations of candidate therapies for alcohol use disorder [80, 81, 82, 83, 84]. Additional applications include studies of polypharmacy and potentially inappropriate medication use [85, 86, 87, 88, 89], antiviral therapies [90, 91], and prescription opioid use trajectories [62]. Importantly, many of these studies have demonstrated consistency with findings from randomized trials [77] and replication across independent cohorts [15, 16, 17, 18, 19], supporting the validity of real‐world evidence generated from this platform.

## 13 Conclusions

VACS‐National is a large, longitudinal real‐world data resource that provides comprehensive clinical, pharmacy, laboratory, and patient‐reported data across a diverse population of Veterans within an integrated healthcare system. Its combination of scale, clinical depth, and reproducible analytic infrastructure supports a wide range of pharmacoepidemiologic applications and the generation of robust real‐world evidence to inform clinical, regulatory, and public health decision‐making.

## Funding

VACS‐National is supported by the National Institute on Alcohol Abuse and Alcoholism (P60‐AA032190, P01‐AA029545, U01‐AA026224, U24‐AA020794, U01‐AA020790, U10‐AA013566). VCM received support from the Emory Center for AIDS Research (P30 AI050409). The funders had no role in considering the study design or in the collection, analysis, interpretation of data, writing of the report, or decision to submit the article for publication.

## Ethics Statement

VACS‐National was approved by the institutional review boards of Yale University (ref #1506016006) and VA Connecticut Healthcare System (ref #AJ0013).

## Consent

VACS‐National has been granted a waiver of informed consent and is compliant with the Health Insurance Portability and Accountability Act.

## Conflicts of Interest

Vincent C. Marconi has received investigator‐initiated research grants (to the institution) and research support from Eli Lilly, Bayer, Gilead Sciences, Merck, Pfizer, Optinosis, and ViiV. No other conflicts are declared.

## Data Availability

Due to VA regulations and data governance requirements, analytic datasets derived from VACS‐National are not permitted to leave the VA secure computing environment without an approved data use agreement. However, VA data are made available to researchers with an approved VA study protocol. Access requires appropriate regulatory approvals, including VA research and development review, and must be conducted within secure environments such as VINCI. For more information, please visit https://www.virec.research.va.gov. Researchers interested in discussing potential collaborations or research opportunities using VACS‐National may contact the VACS team at vacs@yale.edu or visit https://medicine.yale.edu/internal‐medicine/vacs/.
